# Transition Metals (Cr^3+^) and Lanthanides (Eu^3+^) in Inorganic Glasses with Extremely Different Glass-Formers B_2_O_3_ and GeO_2_

**DOI:** 10.3390/ma14237156

**Published:** 2021-11-24

**Authors:** Karolina Kowalska, Marta Kuwik, Justyna Polak, Joanna Pisarska, Wojciech A. Pisarski

**Affiliations:** Institute of Chemistry, University of Silesia, Szkolna 9 Street, 40-007 Katowice, Poland; justyna.polak@us.edu.pl (J.P.); joanna.pisarska@us.edu.pl (J.P.); wojciech.pisarski@us.edu.pl (W.A.P.)

**Keywords:** glasses, network-former, luminescence properties, red-emitting materials, paramagnetic ions

## Abstract

Glasses containing two different network-forming components and doped with optically active ions exhibit interesting properties. In this work, glass systems based on germanium dioxide and boron trioxide singly doped with lanthanides (Eu^3+^) and transition metals (Cr^3+^) ions are research subjects. Optical spectroscopy was the major research tool used to record excitation and emission spectra in a wide spectral range for studied systems. The emitted radiation of glasses doped with Cr^3+^ ions is dominated by broadband luminescence centered at 770 nm and 1050 nm (^4^T_2_ → ^4^A_2_). Interestingly, the increase of concentration of one of the oxides contributed to the detectable changes of the R-line (^2^E → ^4^A_2_) of Cr^3+^ ions. Moreover, EPR spectroscopy confirmed the paramagnetic properties of the obtained glasses. The influence of molar ratio GeO_2_:B_2_O_3_ on spectroscopic properties for Eu^3+^ ions is discussed. The intensity of luminescence bands due to transitions of trivalent europium ions as well as the ratio R/O decrease with the increase of B_2_O_3_. On the other hand, the increase in concentration B_2_O_3_ influences the increasing tendency of luminescence lifetimes for the ^5^D_0_ state of Eu^3+^ ions. The results will contribute to a better understanding of the role of the glass host and thus the prospects for new optical materials.

## 1. Introduction

In recent years, the field of engineering materials (including glasses) has evolved, introducing new information about the interesting properties of systems [1,2,3]. Glass is a material characterized by a lack of long-range order with no defined glass transition temperature [4]. In an excellent communication [5], Zanotto and Mauro declared that a comprehensive definition of glass, combining the aspect of knowledge improvement and modernity, is still being explored. Interestingly, to this day, the technology to prepare glass is based on substances such as SiO_2_, B_2_O_3_, GeO_2_, P_2_O_5_, which are the only ones that meet Zachariasen’s rules for glass formation [6]. A variety of modifications in the chemical composition of glasses enables the introduction of different optically active ions such as transition metal and rare-earth ions. This allows obtaining unique luminescent properties and developing new optical materials operating in a wide spectral range. The glass systems are applied in light-emitting diodes, lasers, phosphors, or optical amplifiers [7,8,9]. In defining the spectroscopic properties of amorphous materials, a significant contribution is the composition of the glass matrices. In particular, the type and concentration of the network-former and/or network-modifier influence structural [10,11], thermal [12], and optical properties [13,14,15]. Recently, Jiao et al. [16] presented that increasing the boron oxide ratio visibly improves the luminescence characterizations of Sm^3+^, Dy^3+^, and Tb^3+^ ions in glass systems. However, the wide range of compositional modifications presented for silicate glasses containing different network modifier oxides [17] indicated significant differences in the local surroundings of Eu^3+^ ions. Very satisfactory results of analysis of luminescence decay kinetics were also obtained for phosphate glasses. The value of measured lifetime for ^4^F_3/2_ level of Nd^3+^ ions increased to 2.49 ms with changing the glass composition [18]. 

The number of publications indicates that among various inorganic systems, borate glasses are preferred for the luminescent centers due to their spectroscopic properties [19]. Divina et al. [20] carried out spectroscopic studies of alkali lead-bismuth borate glasses doped with Dy^3+^ ions. It was reported that these systems exhibit yellowish-white luminescence and suggested the utility for w-LED applications. Borate glasses can also be useful for infrared emitting device applications due to intense near-infrared emission at 1056 nm corresponding to the ^4^F_3/2_ → ^4^I_11/2_ transition of Nd^3+^ ions [21]. However, these glasses are characterized by high phonon energy (1300–1500 cm^−1^), and it could have a negative effect on the photoluminescence properties of systems doped with rare-earth ions [22]. On the other hand, it limits their application potential in photonic applications. Therefore, it is preferable to introduce other oxides to the host matrix to reduce the probability of non-radiative transitions. Germanate glasses have been of great interest for many years because of the specificity of their physical properties, such as glass transition temperature, particularly [23]. Moreover, germanate glass systems exhibit the ability to absorb X-rays and high transparency in the near-infrared spectral region [24]. These systems have unique properties such as low phonon energy (~800 cm^−1^), good thermal stability, and good lanthanide ions solubility [25]. The results obtained in our previous works [26] proved that the developed glasses containing germanate (IV) oxide and titanate (IV) oxide determine the differences in the profile of the registered luminescence bands of d-transition metal and rare-earth ions. The present paper presents the correlation between the content of two different glass-network formers (GeO_2_ and B_2_O_3_) and the spectroscopic properties of inorganic glasses singly doped with transition metal ions (Cr^3+^) and lanthanide ions (Eu^3+^). 

The spectroscopic properties of rare-earth and transition metal ions in materials are subject to rigorous interpretation that provides important contributions to contemporary scientific investigations. The luminescence spectra of Eu^3+^ ions are characterized by f–f transitions [27,28], respectively. The first observation of these transitions dates from 1901 by Demarcay, considered the discoverer of the europium ion [29]. According to the work of Binnemans [30], the relative intensities of transitions in emission spectra can be used to probe the local environment around Eu^3+^ ions. Analysis of the properties of glass systems can also be spectroscopically monitored by trivalent chromium ions [31]. Modification of the quantitative relationship of glass-former and glass-modifier oxides causes them to occupy different sites with different crystal field strengths [32,33]. Moreover, the laser transition ^4^T_2_ → ^4^A_2_ of Cr^3+^ ions is very sensitive to its chemical environment.

Based on these criteria, a series of glasses with the following chemical formula GeO_2_-B_2_O_3_-BaO-Ga_2_O_3_-Cr_2_O_3_ and GeO_2_-B_2_O_3_-BaO-Ga_2_O_3_-Eu_2_O_3_ were synthesized. The glass systems were obtained using the conventional high-temperature melt-quenching technique of high-purity metal oxides as starting materials. The selected optically active ions have a special role in the research because they are useful spectroscopic probes. For this reason, the structural properties of glasses doped with Cr^3+^ ions were investigated using electron paramagnetic resonance spectroscopy. The luminescence characterization of this study was aimed at determining which of the glass-network oxides favorably influence the optical properties of the obtained glasses. Excitation and emission spectra in the visible and near-infrared ranges were recorded. In particular, spectroscopic features such as R-line luminescence, superimposed on the broad emission band ^4^T_2_ → ^4^A_2_ transition of chromium ions, were analyzed. On the other hand, a fluorescence intensity ratio (R/O) parameter as a function of GeO_2_:B_2_O_3_ concentration was estimated of ^5^D_0_ → ^7^F_J_ (J = 1–2) transitions of europium ions. Moreover, luminescence lifetimes for the upper ^5^D_0_ excited level of Eu^3+^ ions are evaluated and discussed.

## 2. Materials and Methods

### 2.1. Glass Synthesis

In the present work, a series of inorganic glasses were synthesized using a high-temperature melt quenching-technique. The appropriate amounts of anhydrous metal oxides: germanium (IV) oxide (Sigma-Aldrich Chemical Co., St. Louis, MO, USA, ≥99.99%), boron trioxide (Sigma-Aldrich Chemical Co., St. Louis, MO, USA, 99.98%), barium oxide (Sigma-Aldrich Chemical Co., St. Louis, MO, USA, 99.99%), gallium (III) oxide (Sigma-Aldrich Chemical Co., St. Louis, MO, USA, >99.99), chromium (III) oxide (Sigma-Aldrich Chemical Co., St. Louis, MO, USA, 99.9%), europium (III) oxide (Sigma-Aldrich Chemical Co., St. Louis, MO, USA, 99.99%), were carefully homogenized in an agate mortar. Melting of the desired mixtures in corundum crucibles was carried out in an electric furnace in an air atmosphere at the temperature of 1250 °C. The glass samples were kept at this temperature for 1 h before slowly cooled down to room temperature. Then the samples were subjected to grinding and polishing treatment. Based on X-ray diffraction measurements it has been confirmed that all samples are fully amorphous. As a result of the above synthesis procedure, two series of glass samples doped with transition metal (Cr^3+^) and lanthanide (Eu^3+^) ions were obtained (Table 1 and Table 2). 

### 2.2. Characterization Techniques

We used the following research tools to characterize the sample properties: electron paramagnetic resonance (EPR) spectroscopy and optical spectroscopy. EPR spectroscopy was used to describe the composition of glasses doped with transition metal ions (Cr^3+^). Each glass sample in powdered form was placed inside a special quartz tube. EPR spectra were recorded using Bruker EMX EPR spectrometer (Bruker-Biospin, Karlsruhe, Germany) operating at X-band frequency (9.8 GHz) with a modulation amplitude of 2.0 G. The magnetic field was scanned from 1000 G to 5000 G. The EPR instrument parameters are as follows: central field 3480 G, time constant 40.96, gain 1 × 10^4^ G, microwave power 20.12 mW. 

The optical properties of glasses were analyzed using luminescence spectroscopy with the system PTI QuantaMaster QM 40 UV/VIS Steady State Spectrofluometer (Photon Technology International, Birmingham, NJ, USA). The laser equipment was coupled with a tunable pulsed optical parametric oscillator (OPO), pumped by the third harmonic of a Nd:YAG laser (Opotek Opolette 355 LD, Carlsbad, CA, USA). The system consisted of a double 200 mm monochromators, a xenon lamp as a light source, a multimode UV-VIS PMT (R928) (PTI Model 914), and Hamamatsu H10330B-75 (Hamamatsu, Bridgewater, NJ, USA) detectors, PTI, and ASOC-10 USB-2500 oscilloscope. Resolution for spectral measurements (excitation and emission spectra) was ±0.25 nm (Cr^3+^-doped glass samples) and ±0.5 nm (Eu^3+^-doped glass samples), whereas decay curves with accuracy 0.5 µs were acquired. All measurements were performed at room temperature. 

## 3. Results and Discussion

### 3.1. Transition Metals—Cr^3+^

**Figure 1:**
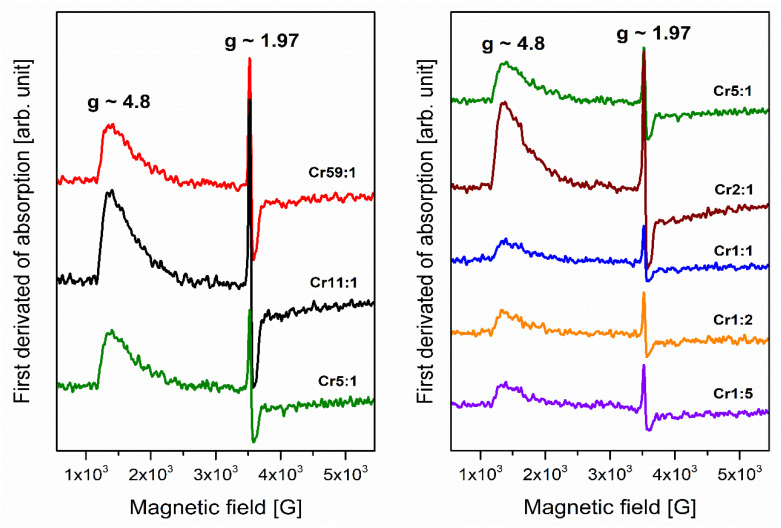
Electron paramagnetic resonance spectra of Cr^3+^ ions doped glass samples with different concentrations of glass-former components (GeO_2_ and B_2_O_3_).

Electron paramagnetic resonance (EPR) is a significant experimental technique for determining the environment of transition metal (Cr^3+^) ions in glasses. Analysis of EPR spectra gives information about the glass network and symmetry around transition metal ions. Figure 1 presents electron paramagnetic resonance spectra of glassy systems containing two types of glass-formers and doped with Cr^3+^ ions. Independently on the molar ratio GeO_2_:B_2_O_3_, the similar registered EPR spectra show one signal at a low magnetic field and one resonance line at a high magnetic field. 

The broad asymmetric signal with g value ~4.8 corresponds to isolated Cr^3+^ centers in strongly distorted octahedral sites [34]. On the other hand, the narrow resonance line with g close to 1.97 is assigned to exchange-coupled Cr^3+^-Cr^3+^ pairs [35], or this signal can be attributed to the trivalent chromium ions in cubic sites of the glass network [36]. The similar measured effective g values obtained for studied glasses were also noticed for Cr^3+^ ions in CdO-SrO-B_2_O_3_-SiO_2_ [37], ZnO-As_2_O_3_-Sb_2_O_3_ [38], and Li_2_CO_3_-B_2_O_3_-P_2_O_5_ systems [39]. 

**Figure 2:**
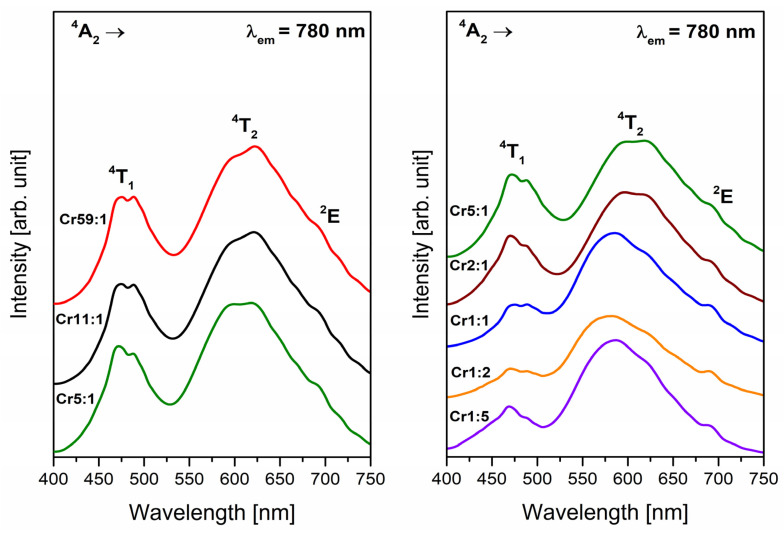
Excitation spectra of Cr^3+^ ions doped glass samples with different concentrations of glass-former components (GeO_2_ and B_2_O_3_) monitored at 780 nm.

The intensity of the signals is higher for glasses with a dominant concentration of glass-former GeO_2_ (samples from Cr59:1 to Cr2:1). However, the intensity of resonance lines is lower for samples with increasing content of glass-former B_2_O_3_. Apart from the variation in signal intensity, no important modifications were observed when the glass-formers concentrations in glass composition changed. The influence of glass-formers on the spectral properties of systems doped with Cr^3+^ ions was studied. To this goal, the excitation spectra were monitored at λ_em_ = 780 nm (Figure 2), and two broad bands were observed due to transitions from the ^4^A_2_ ground level of trivalent chromium ions. From the literature, it is well known that Cr^3+^ ions have strong visible absorption due to the spin-allowed but parity-forbidden transitions [32].

The first excitation band around at 480 nm corresponds to the transition ^4^A_2_ → ^4^T_1_ of Cr^3+^ ions. In contrast, the second band consists of three overlapped peaks located at 600 nm, 625 nm, 690 nm and related to transitions originating from ground level to the ^4^T_2_, ^2^T_1_, and ^2^E excited levels of Cr^3+^ ions, respectively. It is worth noting that the intensity of band maxima of the ^4^A_2_ → ^4^T_2_ and ^4^A_2_ → ^2^T_1_ transitions change with the molar ratio of glass-formers (GeO_2_:B_2_O_3_). The intensity of the band corresponding to the transition ^4^A_2_ → ^4^T_2_ of Cr^3+^ ions increases when the content of germanium dioxide decreases. On the other hand, the intensity of band attributed to excitation level ^2^T_1_ is the highest for system Cr59:1 and the lowest for glass sample Cr1:5. The effect of changing the glass composition on the intensity of excitation bands was also observed for borate glass-ceramics with a constant concentration of trivalent chromium ions [40]. Moreover, the analysis of excitation spectra indicates that the forbidden electron transition ^4^A_2_ → ^2^E of Cr^3+^ ions is more clearly separated for glasses with a higher concentration of boron trioxide as glass-former (Cr1:1, Cr1:2, Cr1:5). To study the luminescence properties of systems with both glass-formers (GeO_2_ and B_2_O_3_), the wavelength λ = 600 nm was chosen for registration emission spectra.

**Figure 3:**
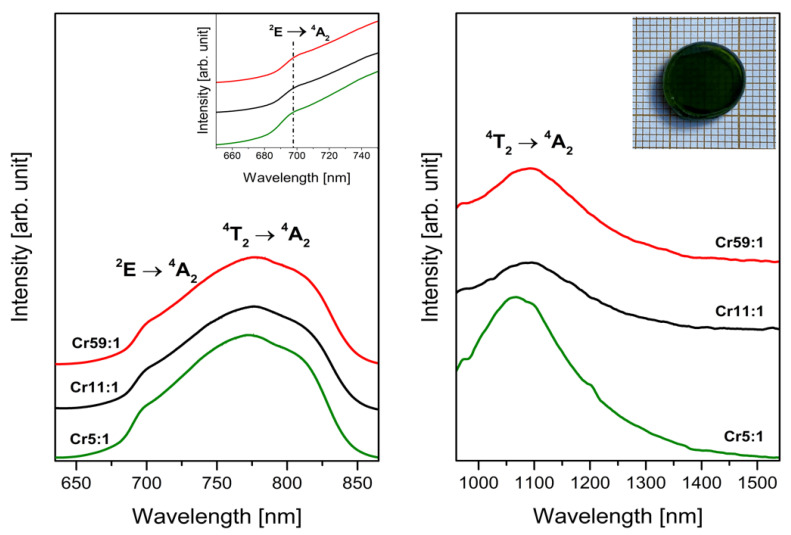
Emission spectra of Cr^3+^ ions doped glass samples (Cr59:1, Cr11:1, Cr5:1) with different concentrations of glass-former components (GeO_2_ and B_2_O_3_). Insets show an enlargement of the emission spectrum corresponding to the R-line and a photographic image of a glass sample doped with Cr^3+^ ions.

**Figure 4:**
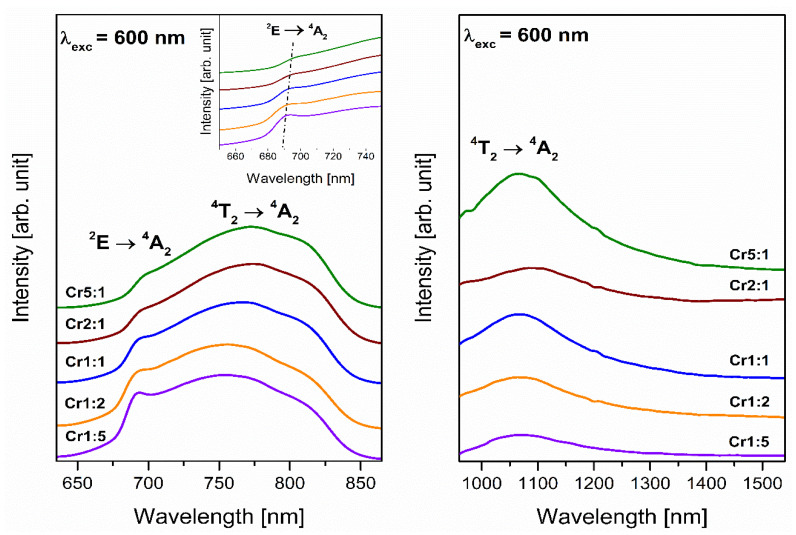
Emission spectra of Cr^3+^ ions doped glass samples (Cr5:1, Cr2:1, Cr1:1, Cr1:2, Cr1:5) with different concentrations of glass-former components (GeO_2_ and B_2_O_3_). The inset is an enlargement of the emission spectrum corresponding to the R-line of Cr^3+^ ions.

Figure 3 and Figure 4 show luminescence spectra registered in the red and near-infrared spectral region. The band located in the range 650–850 nm with a maximum at about 780 nm indicates that all samples exhibit red luminescence irrespective of the molar ratio of glass-formers. The presence of this broad emission band confirms results from analysis of EPR spectra and proves that trivalent chromium ions are in octahedral sites [41]. It is interesting to see that the band is a result of overlapping two transitions of Cr^3+^ ions: allowed ^4^T_2_ → ^4^A_2_ and forbidden ^2^E → ^4^A_2_. The broad band corresponds to allowed transition, and it is attributed to the Cr^3+^ centers in low-field sites [42]. However, the narrow emission band corresponding ^2^E → ^4^A_2_ transition called R-line suggests that chromium ions are subjected to high-field sites [43]. The appearance of the two luminescence bands proves the existence of both octahedral sites. Therefore, some of the Cr^3+^ ions emit from the ^4^T_2_ level and some from the ^2^E level [44]. 

The shape of emission resulting from trivalent chromium ions occupying different sites was changed with molar ratio GeO_2_:B_2_O_3_. The intensity of R-line increases with decreasing concentration of glass-former GeO_2_ in studied systems. It was clearly observed for sample Cr5:1 with weak R-line and sample Cr1:5 with intense narrow emission band (Figure 4). The region 680–700 nm, where the R-line is situated, shows that the spectral lines are slightly shifted toward lower wavelengths with increasing B_2_O_3_ concentration. Simultaneously, the intensity of the broad band related to transition originating from excited level ^4^T_2_ to the ground level ^4^A_2_ of Cr^3+^ ions decreases with changing of glass-formers concentration. However, it is still the dominant emission for Cr^3+^ ions in studied systems. The blue shift of the maximum of this emission band was also stated (λ_max_ = 773 nm and λ_max_ = 753 nm for Cr59:1 and Cr1:5 sample, respectively). According to Narendrudu et al. [45] changing the intensity of transitions ^4^T_2_ → ^4^A_2_ and ^2^E → ^4^A_2_ of trivalent chromium ions can be related to the energy transfer between isolated Cr^3+^ centers and coupled Cr^3+^-Cr^3+^ pairs. Furthermore, Yang et al. [46] reported that the substitution of BaF_2_ in germanate glasses causes enhance broad emission attributed to the ^4^T_2_ → ^4^A_2_ transition of trivalent chromium ions. Our results indicate that luminescence emitted by Cr^3+^ ions in studied systems depends on glass composition. Especially, different glass-formers influence the shape and intensity of emission corresponding to transitions of Cr^3+^ ions. It can be concluded that low-field sites are occupied by the greater part of trivalent chromium ions in samples Cr59:1, Cr11:1, Cr5:1, and Cr2:1 than in glasses with higher B_2_O_3_ concentration. On the contrary, more Cr^3+^ ions are located in high-field sites in glass systems Cr1:2 and Cr1:5 than in samples with dominant content of GeO_2_ as glass-former.

Near-infrared spectra for glass samples with two glass-formers present one luminescence band in the 1000–1400 nm spectral region. Similar emission was observed for lithium, and lithium potassium borate systems and the broad registered band was assigned to transition from the ^4^T_2_ level to the ^4^A_2_ ground level of trivalent chromium ions [47]. Previously published results by Li et al. [48] indicate the luminescence centered at about 1030 nm corresponds to the ^4^T_2_ → ^4^A_2_ transition in tetrahedral sites of Cr^3+^ ions. For that reason, we can conclude that both emission bands with maxima at about 780 nm and 1070 nm are related to the same transition of trivalent chromium ions. However, red luminescence is attributed to Cr^3+^ ions in octahedral sites, and near-infrared emission corresponds to trivalent chromium ions in tetrahedral sites. The molar ratio GeO_2_:B_2_O_3_ influences the intensity of emission but significant differences in the shape of spectra were not observed.

### 3.2. Lanthanides—Eu^3+^

**Figure 5:**
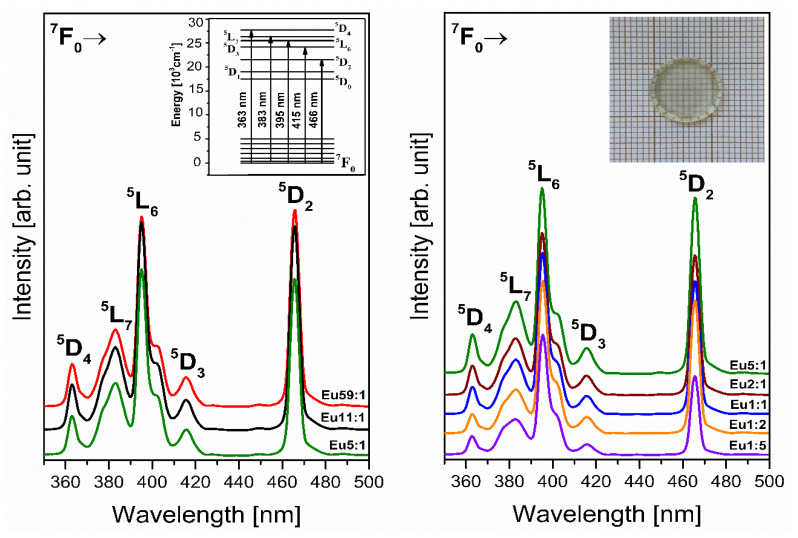
Excitation spectra of Eu^3+^ ions doped glass samples with different concentrations of glass-former components (GeO_2_ and B_2_O_3_) monitored at 611 nm. Insets show the energy level diagram of trivalent europium ions and a photographic image of a glass sample doped with Eu^3+^ ions.

Next, we undertook the characterization of the optical properties for the obtained glass samples doped with europium ions by recording the excitation spectra shown in Figure 5. The excitation spectra were registered in the spectral range from 350 to 500 nm and monitored at 611 nm, the wavelength corresponding to the red emission of the ^5^D_0_ → ^7^F_2_ transition of Eu^3+^ ions. 

The spectra show the occurrence of bands associated with typical 4f^6^–4f^6^ intra-configuration electronic transitions of trivalent europium ions, which can be attributed to individual transitions from the ground state ^7^F_0_ to higher excited states of Eu^3+^ ions [49]. In the analyzed spectral range, bands centered at 363 nm (^7^F_0_ → ^5^D_4_), 383 nm (^7^F_0_ → ^5^L_7_), 396 nm (^7^F_0_ → ^5^L_6_), 415 nm (^7^F_0_ → ^5^D_3_), and 466 nm (^7^F_0_ → ^5^D_2_), were recorded. Preliminary observations of the excitation spectra clearly demonstrated that the intensity of individual bands depends on the molar ratio GeO_2_:B_2_O_3_. By considering the three glass samples Eu1:5, Eu5:1, and Eu59:1, it was concluded that increasing the germanium dioxide content contributes to a gradual increase of the intensity. However, among registered excitation bands, the most intense correspond to ^7^F_0_ → ^5^L_6_ (396 nm) and ^7^F_0_ → ^5^D_2_ (466 nm) transitions of trivalent europium ions, regardless of the molar ratio GeO_2_:B_2_O_3_. According to the results obtained for glasses based on germanium dioxide, the intensity ratio of these excitation bands significantly depends on the glass composition. Ramesh et al. [50] showed that in germanate glasses with PbO the bands related to ^7^F_0_ → ^5^L_6_ and ^7^F_0_ → ^5^D_2_ transitions of Eu^3+^ ions are equally intense. On the other hand, for the same glass systems where PbO was replaced by Bi_2_O_3_, the intensity of the band located at 464 nm (^7^F_0_ → ^5^D_2_) is two times stronger than that centered at 393 nm (^7^F_0_ → ^5^L_6_). Obtained results indicate that the intensity of the excitation bands attributed to ^7^F_0_ → ^5^L_6_ and ^7^F_0_ → ^5^D_2_ transitions of Eu^3+^ ions is similar for system Eu59:1. In contrast, the increase of concentration of boron trioxide causes the decreasing intensity of the band corresponding to the ^7^F_0_ → ^5^D_2_ transition. Additionally, as a function of the concentration of the glass-formers, any spectral shifts were not observed. It is well known that the sharp excitation wavelength gives the intense emission. Hence, bands related to the ^7^F_0_ → ^5^L_6_ and ^7^F_0_ → ^5^D_2_ transitions suggest a potential for effective optical excitation of amorphous materials containing Eu^3+^ ions. For this reason, we chose a wavelength λ_exc_ = 396 nm in the luminescence investigations, to demonstrate the suitability of the obtained glasses for lighting applications.

**Figure 6:**
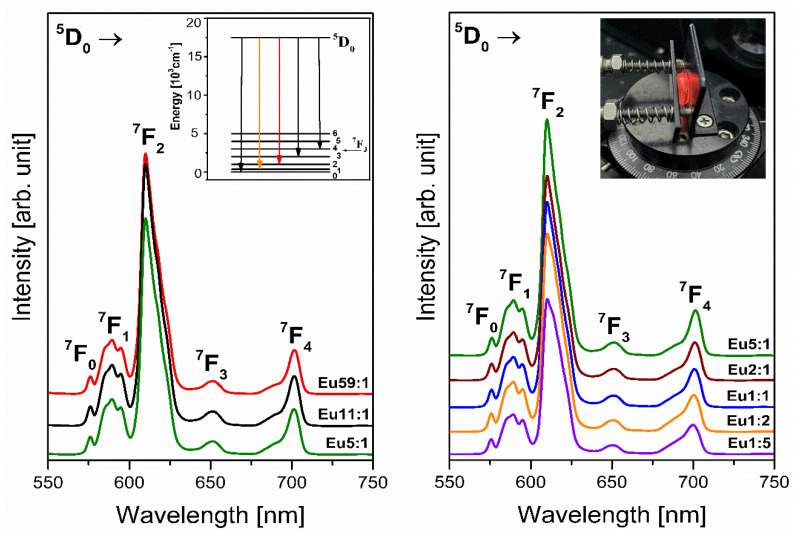
Emission spectra of Eu^3+^ ions doped glass samples with different concentrations of glass-former components (GeO_2_ and B_2_O_3_). Insets present the emission transitions from the excited ^5^D_0_ level of lanthanide ions (Eu^3+^) and photographic image of glass sample under excitation of 396 nm.

The main role analysis of emission and functional properties of the glasses doped with lanthanides ions is the characterization of the emitted radiation, which results from the electron transitions of Ln^3+^ ions [51]. Noticeably, the most significant point of the studies of systems containing trivalent europium ions is to obtain efficient red emission. It is known that the excitation energy of the Eu^3+^ ions is transferred non-radiatively from excited levels ^5^L_6_, ^5^D_3_, ^5^D_2,_ and ^5^D_1_ to the energy level ^5^D_0_. Then the energy transfers back to the ground state causing characteristic visible emissions corresponding to the ^5^D_0_ → ^7^F_J_ transitions (where J = 0–6) of Eu^3+^ ions [52,53,54]. The emission spectra in the visible range under xenon lamp excitation for the obtained glasses were recorded and five well-separated bands were observed. According to the energy level scheme of Eu^3+^ ions, the registered emission bands presented in Figure 6 correspond to the transitions from the excited level ^5^D_0_ to the lower-lying levels ^7^F_0_, ^7^F_1_, ^7^F_2_, ^7^F_3_, and ^7^F_4_ were assigned using the references [55,56]. The presented spectra demonstrate that the intensity of the luminescence band at 611 nm attributed to the red emission of europium ions increases two times when the molar ratio GeO_2_:B_2_O_3_ increases in the direction from 1:5 to 5:1.

As is generally known, the presence of all emission bands depends on the local centrosymmetry of Eu^3+^ ions in the studied glasses [57]. If trivalent europium ions in glass hosts are in a higher asymmetrical environment, the band related to transition ^5^D_0_ → ^7^F_0_ could be recorded [58]. Moreover, the emission transitions ^5^D_0_ → ^7^F_1_ and ^5^D_0_ → ^7^F_2_, ^5^D_0_ → ^7^F_4_ are allowed by magnetic and electric dipole interactions, respectively. It should be emphasized here that Eu^3+^ ions in the literature represent an attractive active dopant because, in addition to very efficient emission, they play an important role as a sensitive spectroscopic probe [59,60]. Two spectral ranges are relevant from this point of view. 

**Figure 7:**
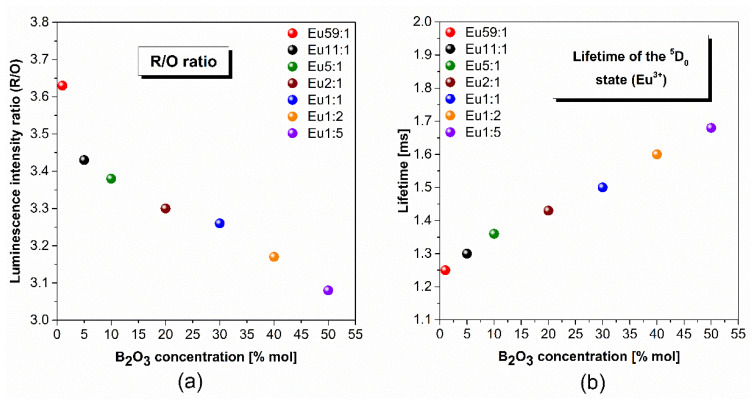
(**a**) Asymmetric ratio and (**b**) luminescence lifetime of obtained glass samples doped with trivalent europium ions containing extremely different glass-formers concentrations.

The first range (580–600 nm) includes the band corresponding to the ^5^D_0_ → ^7^F_1_ transition, and this transition follows the selection rule ∆J = 1. However, the second range (602–636 nm) contains a band associated with the ^5^D_0_ → ^7^F_2_ transition defined as a “hypersensitive” transition because the local environment strongly influences it and this transition of Eu^3+^ ions follows the selection rule ∆J = 2. Similar to the excitation spectra, the concentration of germanium dioxide is a critical factor determining the intensity of the narrow emission band associated with the ^5^D_0_ → ^7^F_2_ transition. This indicates that its intensity depends on the distortion (asymmetry) of the Eu^3+^ coordination polyhedron. The intensity of the band corresponding to the ^5^D_0_ → ^7^F_1_ transition is usually independent of the environment of the Eu^3+^ centers, which is also confirmed by the recorded emission spectra. For this reason, these transitions are fundamental in the analysis of the asymmetric ratio R/O = I(^5^D_0_ → ^7^F_2_)/I(^5^D_0_ → ^7^F_1_). Considering the spectral range of these two transitions, the parameter R/O is also called the red-to-orange fluorescence intensity ratio of Eu^3+^ ions. In agreement with the literature, an appropriate selection of the oxides in glass compositions can influence the value of red-to-orange fluorescence intensity ratio of Eu^3+^ ions (Table 3). The reported results inform us about the local structure around the trivalent europium ions and the covalence degree of the Eu^3+^-O^2−^ bond. The low value of the R/O ratio is usually attributed to the higher symmetry of the local environment around the Eu^3+^ ions. In contrast, an increase of the R/O value is due to an increase in the asymmetry of the local environment around the dopant ions [61,62]. The results of the conducted experiment showed an interesting correlation, which is shown in Figure 7a and Table 4.

The R/O ratio of the prepared systems with two glass-formers was found to have a minimum of 3.08 for the glass sample containing the predominant concentration of B_2_O_3_ (Eu1:5). Interestingly, as a function of increasing germanium dioxide concentration, the value of R/O parameter is a maximum of 3.63. Therefore, the local symmetry around the Eu^3+^ ions and ionic character of Eu^3+^-O^2−^ bond increases with the change in the molar ratio GeO_2_:B_2_O_3_ (from Eu59:1 to Eu1:5). The observed differences in the local environment around trivalent europium ions are due to the quantitative relationship of the two different glass-former oxides, which act as a more network-former or network-modifier component depending on the concentration.

Based on luminescence decay curves of the ^5^D_0_ level of Eu^3+^ ions in glass systems with two glass-formers GeO_2_ and B_2_O_3_, the luminescence lifetimes were determined. Another notable observation of the consequence of the change in the molar ratio (GeO_2_:B_2_O_3_) is schematized in Figure 7b and Table 4. In the studied glasses, the luminescence lifetime from upper excitation state ^5^D_0_ of trivalent europium increased in the direction from 1.25 ms to 1.68 ms for samples Eu59:1 and Eu1:5, respectively.

Analyzing the composition of obtained glass systems doped with Eu^3+^ ions, it can be stated that due to a higher concentration of boron trioxide, the lifetime for the ^5^D_0_ level is getting longer. Venkatramu et al. [71] reported that the decrease in the luminescence lifetime of the ^5^D_0_ in borate glasses is attributed to the electronegativities of the modifying oxide metals. Simultaneously, in the case of our study, the value of electronegativity Ge and B are comparable, therefore this factor does not significantly affect the luminescence lifetime. It was found that the observed decreasing tendency of a lifetime of the ^5^D_0_ level of Eu^3+^ ions is the effect of the phonon energy of the glassy matrix. From the literature data it is experimentally proved that when the phonon energy of the matrix is higher, there is a higher probability of non-radiative relaxation multiphonon processes, which may result in a reduction of luminescence lifetime for lanthanide ions [72]. A completely opposite relationship was observed, although systems with B_2_O_3_ as glass-former have higher phonon energy than glasses with GeO_2_. In this case, radiative relaxation from the excited levels of trivalent europium ions is dominant due to the very large energy gap between the ^5^D_0_ and ^7^F_6_ energy states of Eu^3+^ ions (ΔE = 12,500 cm^−1^). Consequently, more phonons are required for non-radiative relaxation multiphonon processes in studied systems with glass-former GeO_2_ than samples with B_2_O_3_. Previous experimental results proved this phenomenon because low-phonon germanate glasses doped with Eu^3+^ ions indicated relatively low luminescence lifetime values, 1.43 ms [73], 1.22 ms [74]. It is worth noting that the same parameter determined for high-phonon borate glasses was evaluated at 2.57 ms [75], 2.25 ms [76]. Moreover, the variations in the spectroscopic parameters—the decrease in the R/O ratio and the increase in the lifetimes with increasing B_2_O_3_ and GeO_2_—correlate with the changes in the intensity of the emission bands. The obtained results confirmed the research question of the authors’ investigations and indicated the critical role of glass-former in spectroscopic properties of systems that may find potential use as optical materials.

## 4. Conclusions

In the present work, GeO_2_-B_2_O_3_-BaO-Ga_2_O_3_ glass systems singly doped with transition metals (Cr^3+^) and lanthanides (Eu^3+^) were prepared by high-temperature melt-quenching method. The proposed modification of the concentration of the two main glass-formers components provided the following conclusions:

- Analysis of EPR spectra indicates that the trivalent chromium ions at octahedral sites are present in the glass network. Independently of the molar ratio of different glass-formers, red luminescence was observed for systems doped with Cr^3+^ ions in octahedral sites. The influence of changing concentration glass-formers on the intensity of R-line attributed to Cr^3+^ ions in high-field sites was proved. It was stated that the emission bands with maxima at about 780 nm and 1070 nm are related to the same transition (^4^T_2_ → ^4^A_2_) of trivalent chromium ions in octahedral and tetrahedral sites, respectively.
- Confirmation that the type of glass-formers has a significant contribution to the properties of amorphous materials is presented by the results of spectroscopic studies conducted for glass samples doped which Eu^3+^ ions. Detailed spectroscopic analysis of the emission spectra showed a gradual quenching of the luminescence as a function of boron oxide concentration. The obtained values of the ratio R indicate a more covalent nature of the bond between the lanthanide ions and the surrounding ligands for samples with a higher concentration of germanium dioxide. On the other hand, the increase in boron oxide concentration results in an increase in the values of luminescence lifetimes of ^5^D_0_ level of Eu^3+^ ions.

In summary, the results presented in this manuscript demonstrate that spectroscopic properties of systems doped with transition metal and lanthanides ions should be controlled by the choice of glass-formers. The developed oxide glasses emit efficient radiation in the visible and near-infrared range. From the application point of view, the prepared glasses can be classified as interesting and useful optical materials for potential application in the field of photonics.

## Figures and Tables

**Table 1:** Chemical compositions (mol%) of glass samples doped with transition metal (Cr^3+^) ions.

Sample Code	GeO_2_	B_2_O_3_	BaO	Ga_2_O_3_	Cr_2_O_3_
Cr59:1	59	1	30	9.75	0.25
Cr11:1	55	5	30	9.75	0.25
Cr5:1	50	10	30	9.75	0.25
Cr2:1	40	20	30	9.75	0.25
Cr1:1	30	30	30	9.75	0.25
Cr1:2	20	40	30	9.75	0.25
Cr1:5	10	50	30	9.75	0.25

**Table 2:** Chemical compositions (mol%) of glass samples doped with lanthanides (Eu^3+^) ions.

Sample Code	GeO_2_	B_2_O_3_	BaO	Ga_2_O_3_	Eu_2_O_3_
Eu59:1	59	1	30	9.75	0.25
Eu11:1	55	5	30	9.75	0.25
Eu5:1	50	10	30	9.75	0.25
Eu2:1	40	20	30	9.75	0.25
Eu1:1	30	30	30	9.75	0.25
Eu1:2	20	40	30	9.75	0.25
Eu1:5	10	50	30	9.75	0.25

**Table 3:** R/O-ratio values in various glass matrices.

Glass Composition [mol%]	R/O	References
10GeO_2_-50B_2_O_3_-30BaO-9.75Ga_2_O_3_-0.25Eu_2_O_3_	3.08	Present work
59GeO_2_-1B_2_O_3_-30BaO-9.75Ga_2_O_3_-0.25Eu_2_O_3_	3.63	Present work
89.5B_2_O_3_-10Li_2_O-0.5Eu_2_O_3_	2.41	[63]
0.5GeO_2_-63.5SiO_2_-16K_2_O-16BaO-4Eu_2_O_3_	3.46	[63]
84.5GeO_2_-12.5K_2_O-3Eu_2_O_3_	4.60	[63]
49.5BaO-49.5P_2_O_5_-1Eu_2_O_3_	5.28	[64]
30B_2_O_3_-40GeO_2_-29Gd_2_O_3_-1Eu_2_O_3_	3.54	[65]
25Sb_2_O_3_-25GeO_2_-29.2B_2_O_3_-5Al_2_O_3_-10Na_2_O-0.6AgNO_3_-0.2Eu_2_O_3_	2.75	[66]
45P_2_O_5_-45PbO-9Ga_2_O_3_-1Eu_2_O_3_	1.70	[67]
44P_2_O_5_-17K_2_O-9Al_2_O_3_-23PbF_2_-6Na_2_O-1Eu_2_O_3_	2.36	[68]
10Li_2_O-10PbO-7Al_2_O_3_-70B_2_O_3_-3Eu_2_O_3_	2.02	[69]
59.8GeO_2_-15Ga_2_O_3_-25BaO-0.2Eu_2_O_3_	3.72	[70]

**Table 4:** R/O parameter and lifetimes calculated for glass samples doped with Eu^3+^ ions.

Sample Code	GeO_2_:B_2_O_3_ [mol%]	R/O (Eu^3+^)	*τ*_m_ [ms]
Eu59:1	59:1	3.63	1.25
Eu11:1	55:5	3.43	1.30
Eu5:1	50:10	3.38	1.36
Eu2:1	40:20	3.30	1.43
Eu1:1	30:30	3.26	1.50
Eu1:2	20:40	3.17	1.60
Eu1:5	10:50	3.08	1.68

## Data Availability

Not applicable.
